# The First Papillomavirus Isolated from Vulpes vulpes (VvulPV1) Is Basal to the *Gammapapillomavirus* Genus

**DOI:** 10.1128/genomeA.00111-15

**Published:** 2015-03-19

**Authors:** Beatriz Mengual-Chuliá, Ulrich Wittstatt, Ignacio G. Bravo

**Affiliations:** a Infections and Cancer Laboratory, Catalan Institute of Oncology (ICO), Barcelona, Spain; b Bellvitge Institute of Biomedical Research (IDIBELL), Barcelona, Spain; c Department of Animal Diseases, Zoonoses and Infection Diagnostic, Berlin-Brandenburg State Laboratory, Berlin, Germany

## Abstract

We report the complete genomic sequence of Vulpes vulpes papillomavirus type 1 (VvulPV1), isolated from the hair follicles of a red fox. VvulPV1 does not cluster with other carnivoran papillomaviruses, and is instead a sister taxon to the *Gammapapillomavirus* genus, thus sustaining the scenario of a biphasic evolution of papillomaviruses.

## GENOME ANNOUNCEMENT

Papillomaviruses (PVs) are viruses with a circular, double-stranded DNA genome of 7 to 9 kb length. Members of the *Papillomaviridae* infect epithelia in amniotes and are associated with asymptomatic infections, proliferative benign lesions, and different cancers in humans and other animals (1).

A male red fox (*Mammalia*, *Laurasiatheria*, *Carnivora*, *Canidae*, Vulpes vulpes) was found dead, run over in a road accident in Charlottenburg-Wilmersdorf, Berlin, Germany. Veterinary examination showed the animal was free of rabies, echinococcosis, and trichinosis. Some hair follicles from healthy skin were collected, and the DNA extracted tested positive for the presence of a putatively novel PV (2). We have amplified, sequenced, and cloned the full-length genome of the PV in this sample. Sequencing was performed using first 454 (Roche) technology, followed by complete genome resequencing using the Sanger method. We checked for possible contamination with human DNA in the sample by amplification of the b-globin gene. BLAT identified the b-globin sequence obtained (126 pb) to be 98% identical to the corresponding one on chromosome 21 of the dog genome assembly.

The Vulpes vulpes papillomavirus type 1, VvulPV1, genome is 7,519 bp in length and shows classical PV structure: an upstream regulatory region, the early genes E6, E7, E1, and E2, and the late ones, L2 and L1, and misses any E5 gene (3). Maximum likelihood phylogenetic analyses, all using the E1E2, L2L1, or E1E2L2L1 gene concatenates, place VvulPV1 as a basal sister taxon to the *Gammapapillomavirus* (GammaPV) genus, at the nucleotide and amino acid levels. GammaPV is the largest, and still expanding, PV genus (1). This genus includes PVs found exclusively in humans, retrieved from hair follicles, healthy skin and mucosa, and lesions in skin and mucosa. The closest relative to VvulPV1 is HPV180, a member of GammaPV species 10, based on the L1 nucleotide sequence sharing 63% identity. The average identity on the L1 gene of VvulPV1 with all members of the GammaPV genus is 59.2%. Regarding the rest of the genome, the less conserved gene is E6 and the more conserved gene is E1, respectively, sharing on average 47.2% and 60.4% identity at the nucleotide level with other members of the GammaPV genus. Sticking to the present rules for PV classification (4, 5), VvulPV1 should belong to the GammaPV genus, as nucleotide identity to its closest relative based on L1 is marginally above the 60% threshold.

VvulPV1 enlarges the list of PVs isolated from carnivores, within *Canidae*, *Felidae*, *Hyaenidae*, *Mustelidae*, *Otariidae*, Procyonidae, and *Ursidae*. PVs isolated from carnivores are not monophyletic, and appear instead scattered in the PV tree, belonging to the four large PV crown groups. The phylogenetic position of VvulPV1, isolated from other PVs retrieved from carnivores, and being a sister taxon to the GammaPV genus, exclusively encompassing human PVs, further sustains the hypothesis of a biphasic evolution of PVs (6).

### Nucleotide sequence accession number.

The complete genome sequence for VvulPV1 was deposited in GenBank under the accession no. KF857586.
